# Examining evidence of time-dependent treatment effects: an illustration using regression methods

**DOI:** 10.1186/s13063-022-06803-x

**Published:** 2022-10-06

**Authors:** Kim M. Jachno, Stephane Heritier, Robyn L. Woods, Suzanne Mahady, Andrew Chan, Andrew Tonkin, Anne Murray, John J. McNeil, Rory Wolfe

**Affiliations:** 1 Public Health and Preventive Medicine, Monash University, Melbourne, Australia; 2 Clinical and Translational Epidemiology Unit, Massachusetts General Hospital, Boston, MA, USA; 3 Berman Centre for Outcomes and Clinical Research, Hennepin Health Research Institute, Minneapolis, MN, USA; 4 Division of Geriatrics, Department of Medicine, Hennepin County Medical Center and University of Minnesota, Minneapolis, MN, USA

**Keywords:** Time-dependent effects, Proportional hazards, Clinical trials, Flexible parametric modelling, Treatment effect heterogeneity

## Abstract

**Background:**

For the design and analysis of clinical trials with time-to-event outcomes, the Cox proportional hazards model and the logrank test have been the cornerstone methods for many decades. Increasingly, the key assumption of proportionality—or time-fixed effects—that underpins these methods has been called into question. The availability of novel therapies with new mechanisms of action and clinical trials of longer duration mean that non-proportional hazards are now more frequently encountered.

**Methods:**

We compared several regression-based methods to model time-dependent treatment effects. For illustration purposes, we used selected endpoints from a large, community-based clinical trial of low dose daily aspirin in older persons. Relative and absolute estimands were defined, and analyses were conducted in all participants. Additional exploratory analyses were undertaken by selected subgroups of interest using interaction terms in the regression models.

**Discussion:**

In the trial with median 4.7 years follow-up, we found evidence for non-proportionality and a time-dependent treatment effect of aspirin on cancer mortality not previously reported in trial findings. We also found some evidence of time-dependence to an aspirin by age interaction for major adverse cardiovascular events. For other endpoints, time-fixed treatment effect estimates were confirmed as appropriate.

**Conclusions:**

The consideration of treatment effects using both absolute and relative estimands enhanced clinical insights into potential dynamic treatment effects. We recommend these analytical approaches as an adjunct to primary analyses to fully explore findings from clinical trials.

**Supplementary Information:**

The online version contains supplementary material available at 10.1186/s13063-022-06803-x.

## Introduction

The most commonly utilised approach for analysis of time-to-event data in clinical trials is the Cox proportional hazards (PH) model [1]. The advantage of this model is its lack of assumptions about the shape of the underlying hazard functions and presentation of treatment effects on a relative scale as hazard ratios (HRs). Increasingly, trials are being conducted in which the key assumption of PH that underpins this approach and presentation of the treatment effect summarised as being of single fixed magnitude is questionable [2, 3]. Trials of longer duration and larger trials enable investigation of the natural history of the disease and interplay of mechanistic processes over time. They offer compelling rationale for consideration of alternate measures of treatment effect that allow for the examination of non-PH treatment effects over time. Examples of time-dependent (TD) effects include delays until treatment effectiveness as observed in immunotherapy-based oncology trials with minimal benefit in the first few months of treatment followed by a period of effectiveness after the immune system has been activated. In contrast, vaccinations for influenza and whooping cough provide examples of a treatment that is beneficial early after administration but whose effectiveness diminishes over time. Despite the potential importance of TD treatment effects, detailed assessment and reporting of the PH assumption required to assess the appropriateness of presented time-fixed trial results has been less than optimal [4–6].

Parametric models that make assumptions about the shape of the underlying hazard function can be used as an alternative to the Cox model. Models based on the Weibull and gamma distributions can specify increasing, decreasing and inverted hazard functions. However, these models may fail to capture more complex hazard function. A flexible parametric model (FPM) uses spline functions to model the underlying hazard function of any shape or complexity with the advantages of modelling within a regression-based framework [7]. Specifying the baseline hazard allows for the direct estimation of relative and absolute effects of treatment in addition to other useful measures such as differences between survival and hazard functions to be estimated. In particular, the use of the restricted mean survival time (RMST) difference between groups as a distribution-free measure of treatment effect has been gaining attention as a valid measure of treatment effect even when nonproportionality is present [8, 9].

In addition to capturing complex hazard functions under PH [10], flexible parametric survival models can be easily extended to assess for TD treatment effects on the cumulative hazard or hazard scales [11, 12]. A second regression-based method to assess for evidence of TD treatment effects involves pseudo-observations—or jackknife estimates—based on the non-parametric Kaplan-Meier (KM) curves. These pseudo-observations are used to create estimates constructed in such a way that their sample mean estimates the parameter of interest at pre-determined times of interest. The effect of covariates may then be modelled with the pseudo-observations as the response variable in generalised linear models (GLMs) with a suitable link function [13, 14].

Heterogeneity of treatment effects is another form of non-PH that can arise in clinical trials. Treatment effect heterogeneity is when different subgroups of a trial population respond differently to treatment. Prior clinical knowledge of potentially strong predictive factors can—and should—be incorporated into the study design and prespecified analysis plans through selection of sufficiently homogeneous populations that can be expected to benefit from the treatment [15, 16]. Subgroup heterogeneity may in itself also be time-dependent; hence, reported averaged treatment effects, even in subgroup analysis, can obscure interesting insights available from the trial [17]. The Food and Drug Administration (FDA) provides guidance to enhance the collection, availability, and analysis of demographic subgroups to provide valuable information to better inform providers and the FDA about the safe and effective use of new therapeutics [18, 19].

The goal of this paper is to examine whether regression-based methods allowing for TD treatment effects can provide additional or new insights. For illustration, we apply the methods to the effects of daily low-dose aspirin in initially healthy older persons using the large community-based ASPirin in Reducing Events in the Elderly (ASPREE) clinical trial. The ASPREE trial aimed to determine if aspirin improved healthy ageing with a primary composite endpoint of death, dementia, or persistent physical disability. Secondary efficacy and safety endpoints were also collected. For some endpoints, event rates were anticipated to substantially increase with ageing. The large number of participants and long duration of the treatment phase of the trial provide an opportunity to assess the evidence for potential TD treatment effects of clinical interest and to investigate any potential interplay between underlying event rates and non-PH. Editorials accompanying the trial findings support the need for ongoing follow-up of the ASPREE participants to more robustly address hypotheses regarding benefits or harms of aspirin on endpoints in this older population, with additional mechanistic studies particularly for cancer incidence and mortality being critical [20–22].

The rest of the paper is structured as follows: in the Methods section, we give a brief introduction to the different methods used. In the Semi-parametric Cox PH model section, we provide further detail of the ASPREE trial and a selection of endpoints chosen to best illustrate the functionality and interpretability of modelling time dependence of treatment effects. In the Parametric Weibull model section, we present the ASPREE results using the methods described. Finally, we provide discussions and recommendations in the Poyston-Parmar flexible parametric models (FPMs) section.

## Methods

We compare four regression-based approaches for the estimation of the summary treatment effect estimated as either a hazard ratio (HR) or a difference in restricted mean survival time ($\Delta$RMST). The HR estimates were obtained from the Cox model, the Weibull model, and the spline-based flexible parametric model (FPM) all under an assumption of PH. The $\Delta$RMSTs were estimated using the FPM PH model, the FPM allowing for time-dependence of treatment effects and from generalised linear modelling of transformed datasets consisting of pseudo-observations, being jackknife estimates of time-to-event observations for a specific pre-designated time interval had there not been censoring present.

### Semi-parametric Cox PH model

Using a Cox proportional hazards model [1], the hazard function for the $i^{th}$ patient can be written as

$$\begin{aligned} h_i(t) = h_0(t)\text {exp}(x_i \beta ) \end{aligned}$$

where $x_i$ represents covariates with regression coefficients $\beta$ (log hazard ratios) to be estimated from the data, and $h_0(t)$ denotes the baseline hazard function or event rate when all of the covariates are equal to zero or at their specified baseline levels.

The Cox PH model treats the baseline hazard function as a nuisance parameter by maximising the partial likelihood function which permits estimation of the regression parameters but not the baseline hazard function. A key assumption of the Cox PH model is that of PH, in that the effect of a covariate remains constant or fixed in magnitude over the entire follow-up. The Cox model can be extended to incorporate non-proportional effects by including an interaction of the covariate(s) of interest with some function of time. Various diagnostics have been proposed to assess the PH assumption including graphical approaches and analysis based on residuals or by including an interaction of a covariate of interest with a function of time [23, 24]. These tests of PH assumption require correct specification of the function of time and often lack power to detect non-proportionality [25] .

### Parametric Weibull model

When non-constant event rates are anticipated, parametric models are an alternative to the Cox model [6]. Undertaking a parametric approach to the analysis of survival data has a number of benefits. By directly modelling the baseline hazard function, measures of absolute risk, as well as relative risk, can be directly quantified with an associated estimate of uncertainty. There are efficiency gains if the baseline hazard is correctly specified in a parametric approach compared to the equivalent semi-parametric approach. The modelling of TD effects in continuous time can be conducted more easily within a parametric framework. In the ASPREE trial, monotonically increasing event rates were anticipated—and observed—for the majority of the endpoints which motivated the use of a Weibull hazard function to model the baseline hazard rate for this work. The estimates of treatment effect from this fixed distributional parametric approach act as a comparator to both the semi-parametric Cox model and the more flexible parametric models described below.

### Royston-Parmar flexible parametric models (FPMs)

Royston and Parmar introduced FPMs that use restricted cubic splines to model transformations of the survival function, most commonly using the log cumulative-hazard function [7, 26] and later extended to the log hazard function [27] as a tool to capture simple and more complex hazard functions under both PH and non-PH scenarios. In this way, the attraction of the Cox model—allowing the shape of the baseline hazard to be free of any distributional assumptions—is still achieved by allowing the basis function of cubic splines to flexibly fit the baseline hazard. Additionally, FPMs attain the efficiency of parametric models for estimation and interpretability, providing both relative and absolute estimates of treatment effect.

FPMs use restricted cubic spline functions to model the transformation of the survival function. Restricted cubic splines are piecewise cubic polynomials joined together at “knots” with smoothing constraints placed on knot joins and a restriction that the spline function is linear beyond the first and last knots to ensure an overall smooth function that is not unduly affected by sparse data. In the general approach, FPMs are implemented on the log cumulative hazard scale using one set of spline variables with predefined knot positions based on evenly spaced centiles of uncensored log survival times, with boundary knots at the minimum and maximum uncensored log survival times. The number of knots used to model the baseline hazard can be guided by clinical input and model selection criteria.

Time-dependent effects were modelled using a different set of spline variables for each covariate of interest, possibly using a different number of knots in potentially different locations than the spline variables used to model the baseline hazard. Defining $\mathbf {k}_0$ to denote the knots for the baseline hazard and $\mathbf {k}_j$ the knots for the $j^{th}$ TD effect with associated parameters $\mathbf {\delta _j}$ when there are *D* covariates with TD effects, the log cumulative hazard model is

$$\begin{aligned} \ln \left\{ H(t|x) \right\} = s\{\ln (t) | \varvec{\gamma }, \mathbf {k}_0 \} + \sum \limits _{j=1}^D s\{\ln (t) | \varvec{\delta }_j, \mathbf {k}_j \}\mathbf {x}_j +\mathbf {x}\varvec{\beta } \end{aligned}$$

In order to assess the complexity required for the baseline hazard for each endpoint of the ASPREE trial, a series of preliminary models were fit with varying numbers of knots considering possible degrees of freedom (df) ranging from one df to five df for the baseline spline function. Comparisons were then made between the models visually and through using the Akaike information criterion and Bayesian information criterion statistics with smaller values preferred. For all endpoints assessed, allowing for one (corresponding to the Weibull distribution) to three df for the baseline hazard resulted in suitably smooth curves without evidence of overfitting. Time dependence of the treatment effect could be captured with either one or two df for the five different endpoints. We utilised a model with three df for the baseline hazard and allowed for two df for any TD treatment effect [10, 28]. This was a compromise between the most parsimonious model for any given endpoint and the clinical utility of fitting the same model to each of the endpoints.

**Fig. 1:**
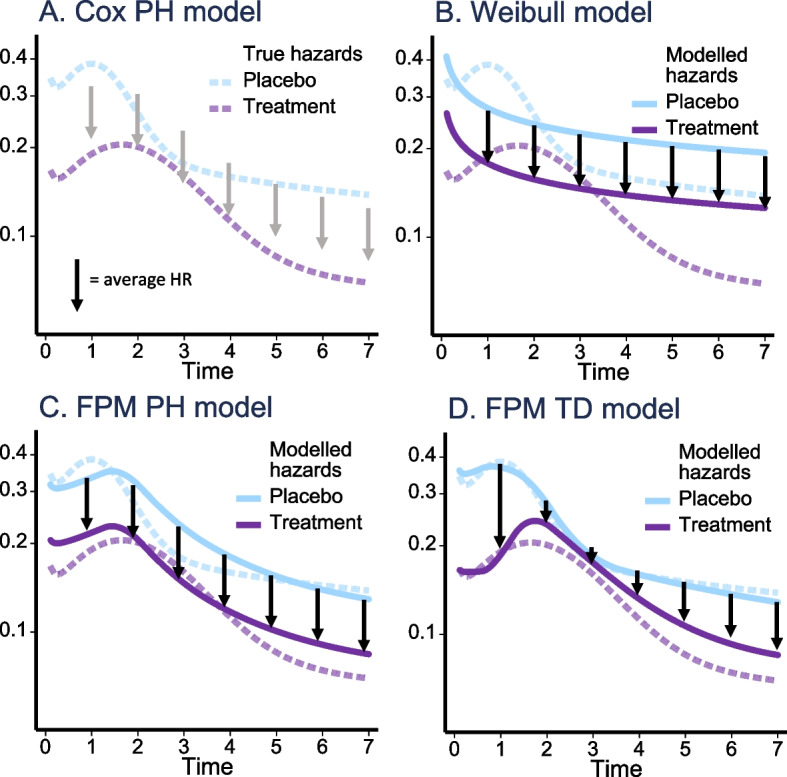
Graphical summary of the regression-based modelling approaches when non-proportional treatment effects are present. Estimated hazards (*y*-axes) and treatment effects from the Cox PH, the Weibull, the FPM PH and FPM TD models when non-proportionality of the true hazards (dashed lines) were present for a hypothetical situation. The arrows indicate the magnitude and direction of treatment effect as measured from the modelled baseline hazard (solid light blue line) to the modelled treatment line (solid purple line)

Figure 1 is a graphical presentation of a hypothetical example where non-proportionality of the treatment effect was present. The true hazard functions (indicated by dashed lines), modelled hazards (solid lines—**b**–**d**), and treatment effects (arrows) in the form of HRs that would arise from application of the Cox PH, the Weibull, and the PH and TD flexible modelling approaches are depicted. The arrows in the Cox PH approach (**a**) represent the constant HR with the absence of solid lines underlining that the hazard function need not be estimated. The solid lines in the Weibull and PH flexible modelling approaches (**b**, **c**) illustrate the constant HR estimated in these approaches. Finally, the varying arrow sizes in the TD flexible modelling approach (**d**) indicate that the estimated treatment effect varies over time, unlike the models represented in **a**–**c**.

### Pseudo-observations approach

Pseudo-observations provide non-parametric estimates of a parameter of interest at the individual participant level [13]. Pseudo-observations are jackknife estimates constructed in such a way that their sample mean estimates the parameter of interest, here the RMST. The pseudo-observations are a transformation of the original data that provides a dataset without censoring. The effect of covariates such as treatment group on the RMST may then be modelled with the pseudo-observations as the outcome variable in GLMs with an appropriate link function. Standard errors of parameter estimates use the robust “sandwich” estimator. The treatment effect estimates of $\Delta$RMST obtained through the pseudo-observations approach are distribution-free since they are based on the KM survival curve estimates and can be used to compare the magnitude of the $\Delta$RMST estimates from the TD FPM. To maintain comparability of the HRs and $\Delta$RMST estimates obtained by the comparator methods, the pseudo-observations approach used the last uncensored event time in the dataset for each endpoint as the time point chosen at which to estimate the mean survival. For analyses of the yearly incremental estimates of treatment effect included as a guide to assessing for non-PH of the main treatment effect, the indicated duration of time was used to estimate the $\Delta$RMST. All statistical analyses were performed with Stata 15 (StataCorp, College Station, TX) with user-written ado file add-ons stpm2 [26], strmst [28], and stpsurv [14].

## The ASPirin in Reducing Events in the Elderly (ASPREE) trial

The ASPREE trial was a community-based randomised trial comparing daily low-dose aspirin versus placebo with the aim of extending the duration of disability-free survival in healthy older adults and was conducted in the US and Australia. Inclusion criteria included ages 70 years or above, except for African-American and Hispanic participants in the USA who were included from age 65 years. Reporting of the ASPREE trial on the primary endpoint and other clinical endpoints utilised a Cox PH modelling approach. This analysis was carried out because the PH assumption was deemed plausible for the primary endpoint components [29–31].

Our analyses were facilitated by the comprehensiveness of data collection in ASPREE, with recruitment of 19,114 participants who attended regular face to face annual study visits for a median of 4.7 years (IQR 3.6–5.7 years). In addition, all major endpoints were adjudicated by Endpoint Committees whose members were blinded to treatment allocation. This enabled us to examine evidence for TD effects of aspirin as well as investigate treatment-covariate interactions of interest. These analyses are to be viewed as supplementary subsidiary analyses to the pre-specified primary analyses already published. Our aim is to illustrate the methods for investigating the magnitude and duration of any treatment effect over time, overall and in specific subgroups of participants even when there was no statistical evidence against the assumption of proportionality.

In this paper, we reexamine the analysis of the primary endpoint of disability-free survival and four other selected endpoints, clinically significant bleeding, major adverse cardiovascular events (MACE), solid tumour cancer incidence, and solid tumour cancer mortality. For each endpoint, we estimate the summary HR treatment effect measure presented previously utilising three different regression-based approaches. In these main analysis models, only the treatment covariate is included to enable comparison with estimates of HR from the unadjusted primary analyses already published. Additionally, we provide the summary $\Delta$RMST treatment effect measure estimated using the same events as for estimation of the summary HR, and graphically display the HR and $\Delta$RMST endpoint measures over time.

### Disability-free survival

Disability-free survival was the primary endpoint of the ASPREE trial. It was a composite endpoint defined as survival free from dementia or persistent physical disability and was derived from the time to first occurrence of any one of the three components of death, dementia, or persistent physical disability in an individual. The endpoint aimed to capture the qualitative and quantitative components of an ongoing healthy life span in an older population considered sufficiently healthy to be enrolled in a primary prevention trial. Details regarding the health measures and definitions used in the trial and the primary conclusion that aspirin use in healthy older adults did not prolong disability-free survival (HR 1.01, 95% confidence interval (CI) 0.92 to 1.11, *p*-value = 0.79) have been reported elsewhere [29].

### Clinically significant bleeding

An increased risk of a clinically significant bleeding event is an adverse effect of aspirin usage [32]. The clinically significant bleeding endpoint of the ASPREE trial included haemorrhagic stroke, symptomatic intracranial bleeding, and clinically significant extracranial bleeding, which were defined as bleeding that led to hospitalisation, prolongation of hospitalisation, surgery, or death. The trial showed the risk of bleeding was significantly higher with aspirin than with placebo (HR 1.38, 95% CI 1.18 to 1.62, *p *< 0.001). The observation of a constantly increasing separation of cumulative incidence curves suggested that the rate of participants newly experiencing bleeding was constant over time [30]. Our analyses further assess and quantify the evidence for persistence of a constant elevated bleeding risk associated with aspirin over the duration of the trial.

### Major adverse cardiovascular events (MACE)

MACE was a non-prespecified composite endpoint which included fatal coronary heart disease (excluding death from heart failure), nonfatal myocardial infarction, and fatal or nonfatal ischaemic stroke. These events were adjudicated as part of the broader cardiovascular disease endpoints, and included the conditions related to ischaemia and atherothrombosis that were anticipated to be affected favourably by low-dose aspirin. The effect of aspirin on MACE events in the trial has been reported previously as a HR of 0.89, 95% CI 0.77, 1.03 [30].

### Solid tumour cancer mortality and incidence

Cancer incidence was a prespecified endpoint in the trial. At the time of the trial’s conception, there was emerging evidence to suggest that low dose regular aspirin usage may be a potential cancer preventative [33]. As participants with a history of cancer were able to enter the trial, incident cancer events included in analysis required diagnosis of new site-specific cancers post randomisation. For the present analysis, only solid tumour cancers were considered in order to be consistent with previous analyses [33]. The effect of aspirin on solid tumour cancer incidence was reported as a HR of 1.05, 95%CI 0.95 to 1.14; the effect of aspirin on cancer mortality was reported as a HR of 1.35, 95%CI 1.13 to 1.61 [34].

Possible time-dependence of these cancer endpoints was acknowledged with additional mechanistic studies and further follow-up called for [21]. We aim to further explore possible time-dependence of treatment effect for the solid tumour cancer endpoints as suggested by progressive separation of the cumulative incidence curves in previous reports [31, 34].

## Results

Table 1 presents results for the two estimands of treatment effect (HR and $\Delta$RMST) for the selected five endpoints. HR estimates were obtained from the Cox PH model, the Weibull model, and the FPM PH model. $\Delta$RMST estimates were obtained from the FPM PH model, the FPM TD model, and the pseudo-observations (p-obs) dataset. The duration of time at which the final summary estimates of HR were assessed extended from time of randomisation to the time of last endpoint event occurrence in the trial dataset. In order to enable comparability, the same time period was used for the estimation of the $\Delta$RMST for each endpoint.

**Table 1:** Summary of the ASPREE trial results for five endpoints using regression-based modelling approaches assuming PH or allowing for TD treatment effects

Endpoint	Estimation	HR (95% CI), *p-*value	Estimation	$\Delta$RMST (95% CI), *p*-value
	model		Model	
**Primary**	Cox PH	1.01 (0.92,1.11), 0.79	FPM PH	− 0.006 (− 0.047, 0.035), 0.79
	Weibull PH	1.01 (0.92,1.11), 0.79	FPM TD	− 0.005 (− 0.046, 0.036), 0.81
	FPM PH	1.01 (0.92,1.11), 0.79	GLM p-obs	− 0.007 (− 0.049, 0.035), 0.75
**MACE**	Cox PH	0.89 (0.77,1.03), 0.12	FPM PH	0.021 (− 0.006, 0.049), 0.13
	Weibull PH	0.89 (0.77,1.03) 0.12	FPM TD	0.021 (− 0.006, 0.048), 0.12
	FPM PH	0.89 (0.77,1.03), 0.12	GLM p-obs	0.021 (− 0.008, 0.050), 0.16
**Clinically**	Cox PH	1.38 (1.18,1.62), $<0.001$	FPM PH	− 0.050 (− 0.075, − 0.026), $<0.001$
**significant**	Weibull PH	1.38 (1.18,1.62), $<0.001$	FPM TD	− 0.052 (− 0.077, − 0.027), $<0.001$
**bleeding**	FPM PH	1.38 (1.18,1.62), $<0.001$	GLM p-obs	− 0.057 (− 0.084, − 0.029), $<0.001$
**Cancer**	Cox PH	1.05 (0.95,1.15), 0.32	FPM PH	− 0.020 (− 0.059, 0.019), 0.32
**incidence**	Weibull PH	1.05 (0.95,1.15), 0.32	FPM TD	− 0.018 (− 0.058, 0.021), 0.36
	FPM PH	1.05 (0.95,1.15), 0.32	GLM p-obs	− 0.024 (− 0.068, 0.020), 0.29
**Cancer**	Cox PH	1.36 (1.13,1.63), 0.001	FPM PH	− 0.032 (− 0.047, − 0.013), 0.001
**mortality**	Weibull PH	1.36 (1.13,1.63), 0.001	FPM TD	− 0.029 (− 0.048, − 0.010), 0.003
	FPM PH	1.36 (1.13,1.63), 0.001	GLM p-obs	− 0.033 (− 0.055, − 0.012), 0.003

For all five endpoints, the summary results presented here for the Cox PH model agree with the previously reported results in the main and follow-up trial publications [29–31, 34, 35]. The three modelling approaches with the underlying PH assumption gave almost identical estimates of the HR. *P*-values from the three PH modelling approaches and across the HR and $\Delta$RMST estimates from the FPM PH model were also similar. There were some differences between the estimates of $\Delta$RMST from the flexible TD and pseudo-observation modelling approaches; however, these were small and unlikely to have any substantive impact on the clinical interpretation of the results. The FPM PH modelling approach provides a link between the HRs and $\Delta$RMSTs, giving a means to relate the magnitude of treatment effect of a relative hazard reduction to an absolute decreased mean survival time on average. As an illustration, for the clinically significant bleeding endpoint, a 38% increased relative risk of bleeding expressed in terms of the $\Delta$RMST could be equivalently expressed as during the trial, a participant randomised to low-dose aspirin would have experienced either a bleeding event or reached the end of the trial without a bleeding event approximately 18 days (0.050 years) sooner on average than a participant randomised to placebo.

**Fig. 2:**
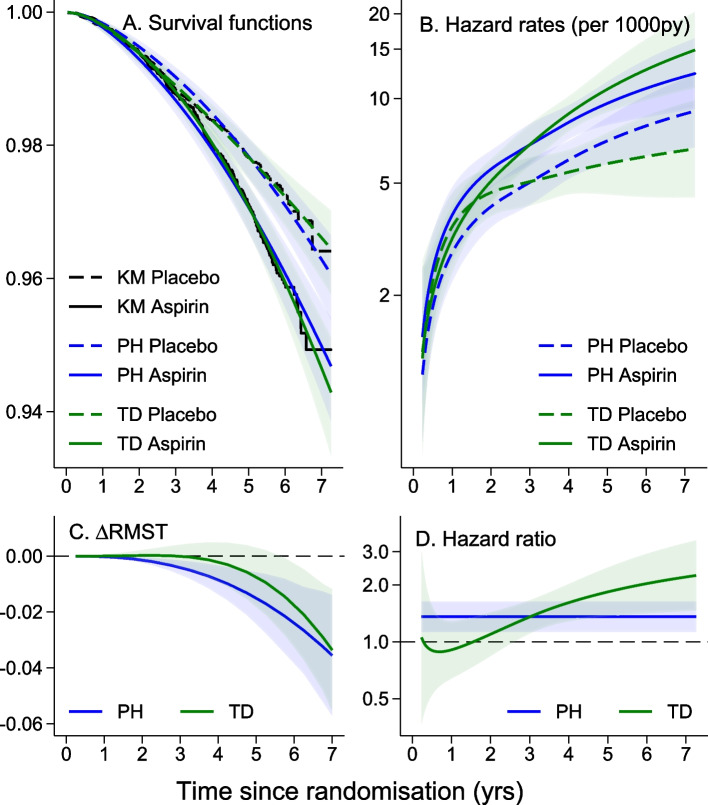
Comparison of PH and TD modelled treatment effects for the cancer mortality endpoint. Survival curves (**A**) and hazard rates (**B**) by treatment arm, and difference in RMST ($\Delta$RMST (**C**)) and HR (**D**) over time from PH (blue curves) and TD (green curves) analysis models for the cancer mortality endpoint. *Y*-axes scales are chosen to emphasis any model or treatment differences

**Fig. 3:**
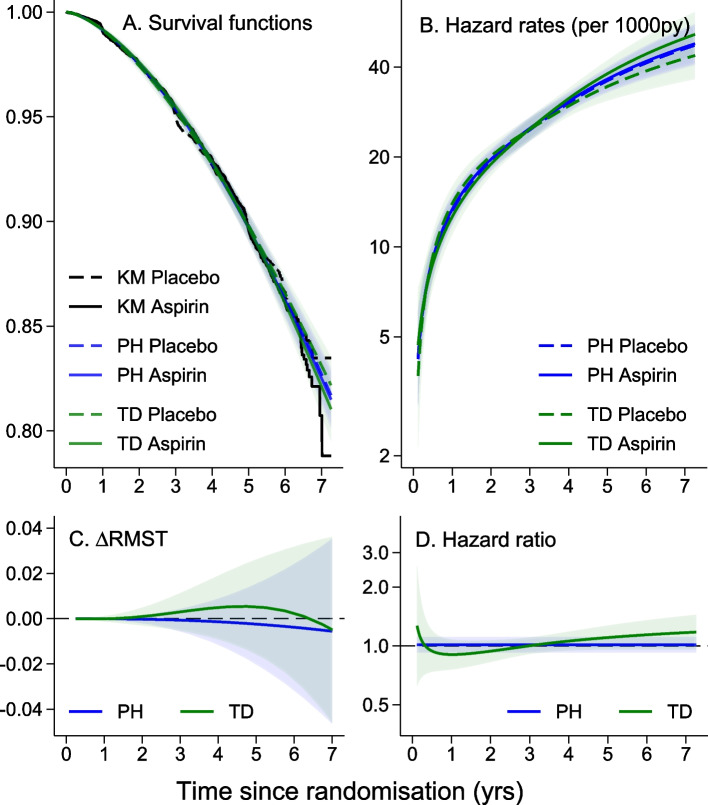
Comparison of PH and TD modelled treatment effects for the composite primary endpoint. Survival curves (**A**) and hazard rates (**B**) by treatment arm, and difference in RMST ($\Delta$RMST (**C**)) and HR (**D**) over time from PH (blue curves) and TD (green curves) analysis models for the composite primary endpoint

For each endpoint, the HR and $\Delta$RMST at yearly incremental durations of time after randomisation are additionally presented in Supplementary Tables S1-S5 available in Additional File 1. These yearly estimates are a tabular subset of the PH and TD analyses of treatment effect presented in C and D of Figs. 2 and 3 (and Supplementary Figs. S1-S3 also available in Additional File 1). Qualitative assessment of TD treatment effects comes from comparing the HRs from yearly incremental durations of follow-up and by comparing the overall HRs with the duration-specific HRs. This is undertaken here regardless of statistical evidence to indicate non-proportionality of treatment effect so caution is warranted with these exploratory analyses to avoid over-interpretation.

Concerning solid tumour cancer mortality, there was an overall increased risk (HR 1.36, 95% CI 1.13, 1.63) found at the end of the trial using a Cox model. However, for this endpoint, there was statistical evidence to indicate non-proportionality of treatment effect (PH test *p* = 0.01 [24]) with the incremental assessments providing some insight into the evolution of this treatment effect. The estimated hazard ratio gradually changed from 0.90 for the first year of the trial (95% CI 0.47,1.73) to 1.20 (95% CI 0.96, 1.50) suggestive of a possible adverse effect of treatment emerging at four years from randomisation (Supplementary Tables S5).

For the major haemorrhage endpoint, there was no statistical evidence to indicate non-proportionality of treatment effect, and although an initial higher treatment-related adverse effect was seen during the first year of follow-up, this stabilised to a lower—but still adverse—effect for the remaining years (Supplementary Table 2). For the primary endpoint, MACE and cancer incidence endpoints, the similarity of the duration-specific HRs over time suggest that a summary estimate of treatment effect was appropriate with little to suggest any time-dependence of effect (Supplementary Tables S1, S3 and S4).

### Exploring time-dependence of treatment effect for the solid tumour cancer mortality endpoint

Figure 2 shows a four-panel graphical presentation of the treatment effect over time for the cancer mortality endpoint. Figure 2A (top left) shows KM survival curves for aspirin and placebo arms, an FPM analysis assuming PH and an FPM analysis allowing for TD of the treatment effect. The KM curves shown in black for the aspirin (solid lines) and placebo (dashed lines) arms in the top left panel (A) show little difference in the first 2–3 years with an apparent separation of the two curves beginning from year 3 onwards. The survival curves from a conventional analysis assuming PH (blue curves) appear to capture the pattern reasonably well. However, even with the greatly expanded *y*-axis used here, differences in the survival proportions can be difficult to discern graphically. The summary HR from the conventional FPM PH model estimates the treatment effect as 1.36 (95% CI 1.13, 1.63; *p* = 0.001) and the $\Delta$RMST to be – 0.032 (– 0.052, – 0.013; *p *= 0.001) indicating worse outcomes in the aspirin arm. The survival curves from the analysis allowing for a TD treatment effect (green curves) are able to capture the lack of separation of the non-parametric KM curves in the first few years of the trial and the increasing separation in the latter years.

The hazard rates by treatment group are presented in Fig. 2B. On this scale, the initial lack of separation of the two groups, followed by a clear separation can be clearly discerned in the curves generated from the FPM allowing for a TD treatment effect. An indication of uncertainty is provided with a shaded 95% CI around the estimated curves. Figure 2C is the difference in RMST ($\Delta$RMST) between the two curves assessed at incremental durations of time since randomisation over the time period 0.25–6.75 years. The emergence of a treatment effect in later years of follow-up is apparent, and it is evident, on the $\Delta$RMST scale, regardless of whether a PH model or a TD model is used. The timing of the emergence of the delayed adverse treatment effect appears to differ between the chosen models. The PH analysis resulted in a larger estimate of treatment effect at all follow-up times considered.

In Fig. 2D, the HR estimates as a function of time since randomisation from the PH and TD analyses of treatment effect are presented. Compared to the summary HR from the PH analysis presented as the constant horizontal line, the HR estimates in the TD analysis varied over time with a gradually increasing harmful effect of aspirin. From a likelihood ratio test of model fit, there is evidence to suggest that the TD model better fits the data compared to the PH model (*p *= 0.03).

### Absence of any time-dependence of treatment effect for the primary and other ASPREE endpoints

An exploratory analysis of treatment effect on disability-free survival, the ASPREE primary endpoint, presented in Fig. 3, shows the survival curves for the aspirin and placebo arms of the trial are almost identical for the entire duration of the trial (A). There was no evidence of a treatment effect and the summary HR estimate of 1.01 (95% CI 0.92, 1.11; *p* = 0.79) or the $\Delta$RMST of – 0.006 (– 0.047, – 0.035; *p* = 0.79) provide an adequate description of the lack of effect of aspirin on this composite outcome over the duration of the trial. Even with an expanded survival proportion axis, the survival proportion curves for the aspirin and placebo arms are almost identical for the entire duration of the trial. The duration of follow-up captured by these analyses is from randomisation until the last uncensored event time in the dataset occurring at 7.01 years.

Similar four panel presentations for the MACE, clinically significant bleeding and cancer incidence endpoints are in Supplementary Figs. S1, S2 and S3 in Additional File 1. For the MACE and cancer incidence endpoints, there is little to differentiate visually between the PH and TD analysis models, confirming the appropriateness of applying single summary estimates of treatment effect for these three endpoints. There is an overall increased risk of clinically significant bleeding due to aspirin with some suggestion that this risk is highest for the first 6 months after commencement of daily usage. This transitory treatment effect is explored further as part of assessing for time-dependent treatment effects by sex (see next section). For all three endpoints, there is no suggestion of improvement of the overall model fit from the likelihood ratio tests comparing the PH and TD approaches.

### Time-dependent treatment effects by subgroup: clinically significant bleeding in males and females

The flexible modelling approaches being examined here can also be used to provide additional insight into interactions between time-dependent treatment effects and subgroups of interest. Here, this is conducted as a post-hoc exploratory analysis although it could form part of a pre-specified analysis plan.

**Fig. 4:**
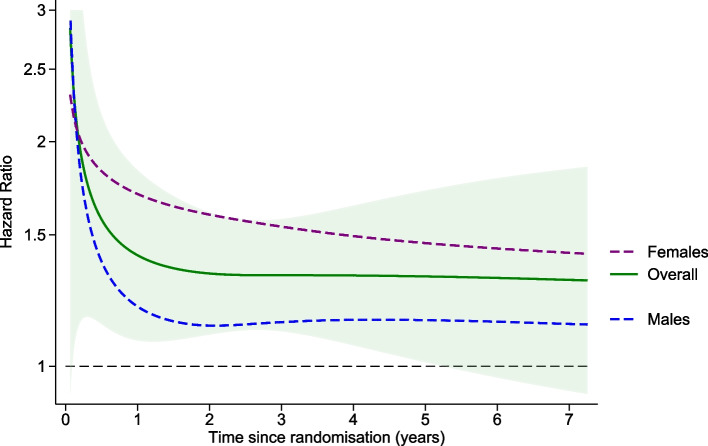
Assessing time-dependence of aspirin treatment for males and females on risk of clinically significant bleeding. The overall estimated HR(t) for treatment effect is the solid green line with the shaded green area indicating the 95% CI width. The HR(t) for treatment effect estimated from females only is indicated by a purple dashed line and the HR(t) for treatment effect estimated from males only indicated by the blue dashed line

For the clinically significant bleeding endpoint, from a comparison of the HR from PH and TD models (see Supplementary Fig. S2 panel D), there is some evidence for an elevated risk in the first year of taking low dose aspirin daily (HR 1.84 95% CI 1.25, 2.70, *p *= 0.002), which then plateaued after the first year to a lower, but still elevated risk (HR 1.30 95% CI 1.08, 1.55, *p *= 0.003) similar to the reported overall HR 1.38 95%CI 1.18, 1.62, *p *< 0.001 for the overall treatment effect from the PH model. Published subgroup analysis by sex did not show strong evidence of different treatment effects in males and females (males HR = 1.21 95% CI 0.97, 1.51; females HR = 1.58, 95% CI 1.26, 1.99; interaction *p*-value = 0.1) [29]. The potential time-dependence of this interaction is explored visually in Fig. 4.

For males, the increased risk of a major bleeding event due to aspirin was at its highest during the first few months although a still-elevated risk persisted throughout the follow-up and was estimated to be approximately constant after the first year of treatment. Compared to males, females had a higher increased risk of bleeding due to daily aspirin usage throughout follow-up. For females, the acute increased risk persisted for most of the first year, and this risk decreased more slowly over the duration of the trial than males. The shaded area in Fig. 4 indicates the uncertainty band around the estimated time-dependent HR for all participants enrolled in the trial and highlights the increasing uncertainty at later timepoints. Supplementary Fig. S4 in Additional File 1 contains graphs for the difference by sex in the HR(t) from the TD analysis for the other four endpoints under consideration.

### Time-dependent treatment effects by subgroup: MACE by age as a continuous covariate

Insight into potential treatment effects and continuous predictor covariates can also be obtained using the FPM approaches. For the ASPREE primary analysis, subgroup effects by age at randomisation were examined categorised as younger than the median age (< 74 years) vs older (74$+$ years) as specified in the statistical analysis plan. For illustration purposes here, in order to maximise power to detect any treatment effect interactions, age was analysed on a continuous scale.

For the MACE endpoint, a tendency towards a greater beneficial treatment effect for the < 74 years age group (HR = 0.76, 95% CI 0.59, 0.97) compared to the 74+ age group (HR = 0.97, 95% CI 0.81, 1.17) has been reported although this interaction was not statistically significant ($\text {p-value}_{\text {int}}= 0.11$) [29]. To illustrate application of the method, age at baseline was included in the PH FPM model as a continuous covariate with an assumed linear association with the endpoint. The evidence of an interaction effect between aspirin and (continuous) age at randomisation was summarised by $\text {p-value}_{\text {int}}= 0.06$. When allowing for TD of the effect of aspirin and age on MACE, the evidence of an interaction effect between aspirin and age was similar $\text {p-value}_{\text {int}}= 0.04$.

**Fig. 5:**
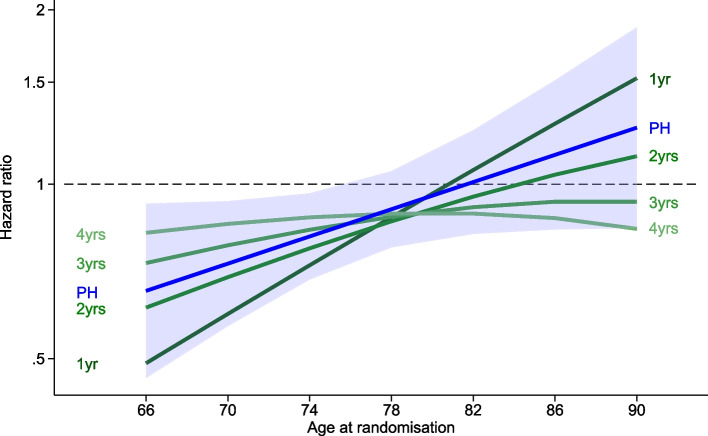
Effect of aspirin on age at randomisation in PH and TD analysis for the MACE endpoint. The estimated age by treatment interaction effect from the PH model is the solid blue line with the shaded area indicating the 95% CI width. The interaction treatment effect from the TD model at yearly intervals is indicated by the green lines with colour intensity decreasing over time

Figure 5 presents these PH and TD FPM analyses assessing treatment effect of aspirin according to age for the MACE endpoint. When a linear relationship between age and MACE was assumed—and one accepts the hypothesis that there is an interaction—the FPM PH analysis showed a protective effect of aspirin at younger baseline ages, increasing towards an absence of any benefit at older ages (blue line with 95% CI shaded area). From the exploratory analysis of the time-dependence of this effect depicted in the green lines in Fig. 5, there is some evidence to suggest that the possible beneficial effect of aspirin for ASPREE participants younger than the median was greatest during the earlier years following randomisation and reduced with time. For participants older than the median, there was no evidence of any benefit of aspirin during the trial. Supplementary Fig. S5 contains graphs of the effect of age with treatment for the other ASPREE endpoints examined in this report. There was no evidence of any interaction effect between aspirin and age in either the PH or TD FPM analyses for these other endpoints.

## Discussion

In this paper, we demonstrated the potential for increased clinical insight using regression-based analysis methods to model the time-dependence of treatment effects compared to methods that assume proportionality of the treatment effect. For five endpoints of the ASPREE trial, we compared the results obtained using the Cox and Weibull PH models to alternative flexible modelling methods utilising splines that are suitable in the context of non-PH and which describe time-dependent treatment effects. We have shown enhanced interpretability by flexibly modelling the baseline hazard or by using the approach of pseudo-observation jackknife estimates in a generalised linear modelling approach. We have further demonstrated the potential of the flexible modelling approaches to explore time-dependent treatment effect heterogeneity in subgroups.

There has been a proliferation of research into analysis methods when non-PH is anticipated or detected with much focus on weighted adaptations to the standard logrank (LR) test in the presence of specific forms of non-pH such as delayed effects [36–41]. Combination tests have also been proposed that combine multiple weighted LR tests and/or weighted LR tests with tests for non-PH designed to provide robust power to detect survival curve differences under a range of non-PH scenarios [42–47]. These hypothesis testing approaches have been aimed at maintaining power to detect statistical significance in clinical trials in the primary analysis. We have focused instead on regression-based approaches and graphical exploratory analyses to examine the evidence for TD treatment effects [48]. In particular, we have utilised the flexible parametric modelling approach as, unlike test-based approaches, it provides estimation of treatment effects under PH and non-PH.

From a clinical perspective, there is utility in being able to present any treatment effects with estimates in both risk-based and time-based metrics which provide complementary information. They provide equivalent information albeit on different metrics when a one-summary treatment effect is sufficient to describe the findings from a trial. When treatment effects vary over time, the different metrics may provide insight into the timing and duration of period specific effects reflective of clinician and patient interest. For three endpoints in the ASPREE trial: disability-free survival, MACE, and cancer incidence, a single HR or $\Delta$RMST provided an appropriate and clinically meaningful summary of the effect of aspirin in healthy older adults, similar in magnitude and direction of treatment effect for the entire duration of the trial. In contrast, for solid tumour cancer mortality and clinically significant bleeding, there was some evidence of time-dependent treatment effects that we now discuss in further detail.

The possible time-dependence of the effect of aspirin on solid tumour cancer mortality suggested adverse effects of treatment emerging by the third year of the trial. We provided evidence that the time-dependent model was a more appropriate fit to the trial data than the proportional hazards model used in the original trial analyses. The findings contrast with the longer-term beneficial effects of aspirin observed in other RCTs. Previously postulated hypotheses to account for this unexpected increase in cancer mortality suggest that the effect of aspirin may have biological effects that vary according to the timing of the exposure, or vary according to age or other participant-specific characteristics. It is conceivable that aspirin may have short-term actions on pathways specific to ageing or tumour cell types in older hosts that could explain the worsened survival among participants in ASPREE in the absence of any apparent effect on cancer incidence [22]. Continued follow-up of ASPREE participants is currently underway to examine legacy effects of the intervention.

For clinically significant bleeding, plausible observations of clinical interest from an analysis of time-dependent treatment effects were seen. An increased risk with aspirin was durable to 5 years of exposure and beyond. There appeared to be a particularly elevated risk of bleeding events with aspirin in the first few months after beginning treatment, which by the end of the first year of follow-up had plateaued to a lower but still increased harmful effect which was then sustained for the remainder of follow-up. However, care is required not to over-interpret this conclusion as the existence of this time-dependence of treatment effect was not confirmed by a statistical test. Hence, clinical and mechanistic plausibility should be considered carefully, and additional studies would be necessary to confirm the working hypotheses regarding any time-dependent aspirin treatment effects.

Further insights into the potential benefits and harms of treatment effects can be demonstrated using flexible modelling approaches by incorporating categorical covariates for subgroups and by allowing continuous covariates to be investigated assuming linear and more flexible spline functional forms. These analyses can provide a more nuanced understanding of potential treatment subgroup heterogeneity and time-dependent treatment effects. Clinical trials are rarely adequately powered to detect interaction effects so any findings need to be considered with the requisite understanding of the exploratory nature of these investigations.

For the clinically significant bleeding endpoint of ASPREE, by allowing for the treatment effect to differ in males and females and allowing that difference to be time-dependent, we were able to demonstrate an acute period of higher risk upon starting daily aspirin usage for both males and females. Our analyses also suggest that females had a relatively higher increased risk of clinically significant bleeding at all times compared to males.

Previous assessments for possible treatment-age interactions for the MACE endpoint in ASPREE had been performed using pre-specified categorical groupings of the age-at-randomisation covariate. Based on the selected categorisations, there had been little evidence to suggest any treatment-age interaction effect (see Supplement S7, S8 in [29]). Our detailed exploratory analysis suggested a beneficial effect of aspirin for ASPREE participants younger than the median age (< 74 years) particularly in the early years of follow-up, but for older participants (74$+$ years), there was no indication of aspirin benefit during the trial.

To more fully report the information in a trial, tabulation of both relative and absolute measures of treatment effect at key times of clinical interest, and graphical presentation of complementary measures of treatment effect over time for subgroups should be encouraged [49]. In this way, readers can ascertain for themselves the suitability of summary treatment effect measures presented as the main findings of the trial. We note that apparent time-dependent treatment effects can arise if underlying event susceptibility varies between participants, a flaw of using relative measures such as the hazard ratio for causal inference [50]. Effect measures directly estimable from absolute risks such as the $\Delta$RMST and difference in survival proportion retain their causal interpretability regardless of the proportionality of the treatment effect and should be used to supplement reports of relative effect measures [51].

## Conclusion

We have compared a range of regression-based approaches allowing for assessment of time-dependent treatment effects and illustrated their potential using a range of endpoints from the ASPREE trial. We recommend these analyses as exploratory and supplementary to the pre-specified primary analyses, aiming to provide enhanced insight and understanding to the mechanisms of any treatment effect, over time and in subgroups of interest. In order to facilitate interpretation, results should be presented using relative and absolute measures of treatment effect in a range of graphical and tabular presentations to provide complementary insights into the timing, magnitude and duration of any treatment effects in a trial.

## Supplementary Information

**Additional file 1:** Supplementary Figures and Tables.

## Data Availability

The datasets used and/or analysed for this publication are available via the ASPREE Principal Investigators. Requests for data access can be directed to aspree.ams@monash.edu. The data are not publicly available due to privacy or ethical restrictions.

## Abbreviations

PH: Proportional hazards
HR: Hazard ratio
TD: Time-dependent
FPM: Flexible parametric model
RMST: Restricted mean survival time
KM: Kaplan-Meier
GLM: Generalised linear model
FDA: Food and Drug Administration
ASPREE: ASPirin in Reducing Events in the Elderly
$\Delta$RMST: Difference in restricted mean survival time
MACE: Major adverse cardiovascular events
p-obs: Pseudo-observations
LR: Logrank
