# Supplementary material for: Examining evidence of time-dependent treatment effects: an illustration using regression methods
Source: Trials. 2022 Oct 6;23:857. doi: 10.1186/s13063-022-06803-x (PMC9535854; doi:10.1186/s13063-022-06803-x)
Supplement: Supplementary file 1 — Additional file 1: Supplementary Figures and Tables. [file 13063_2022_6803_MOESM1_ESM.pdf]

Supplementary material: Examining evidence of time-dependent treatment effects: an illustration using regression methods

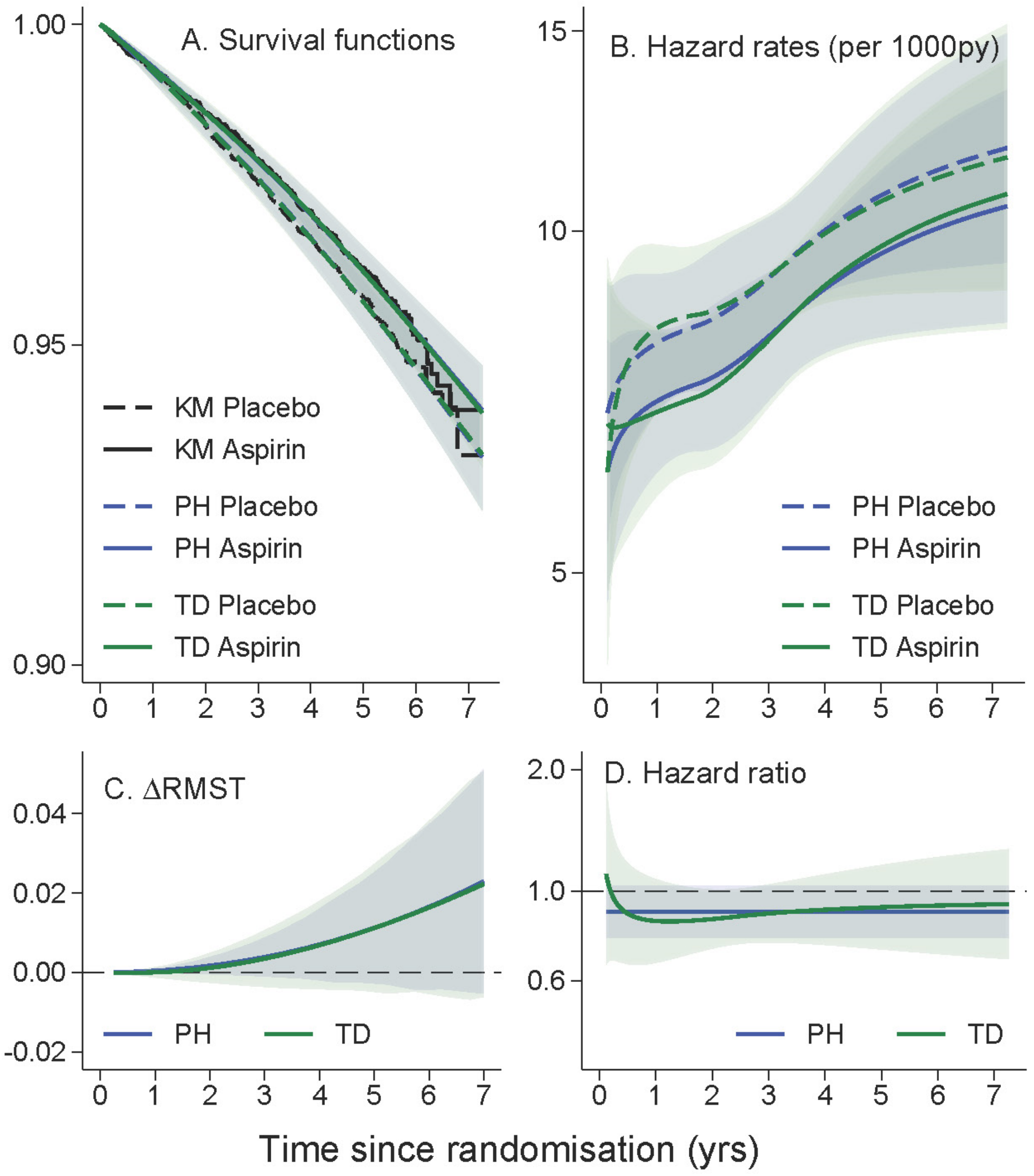

Supplementary Figure S1: Survival curves (panel A) and hazard rates (panel B) by treatment arm, and difference in RMST (ΔRMST; panel C) and HR (panel D) over time from PH and TD analysis models for the MACE endpoint.

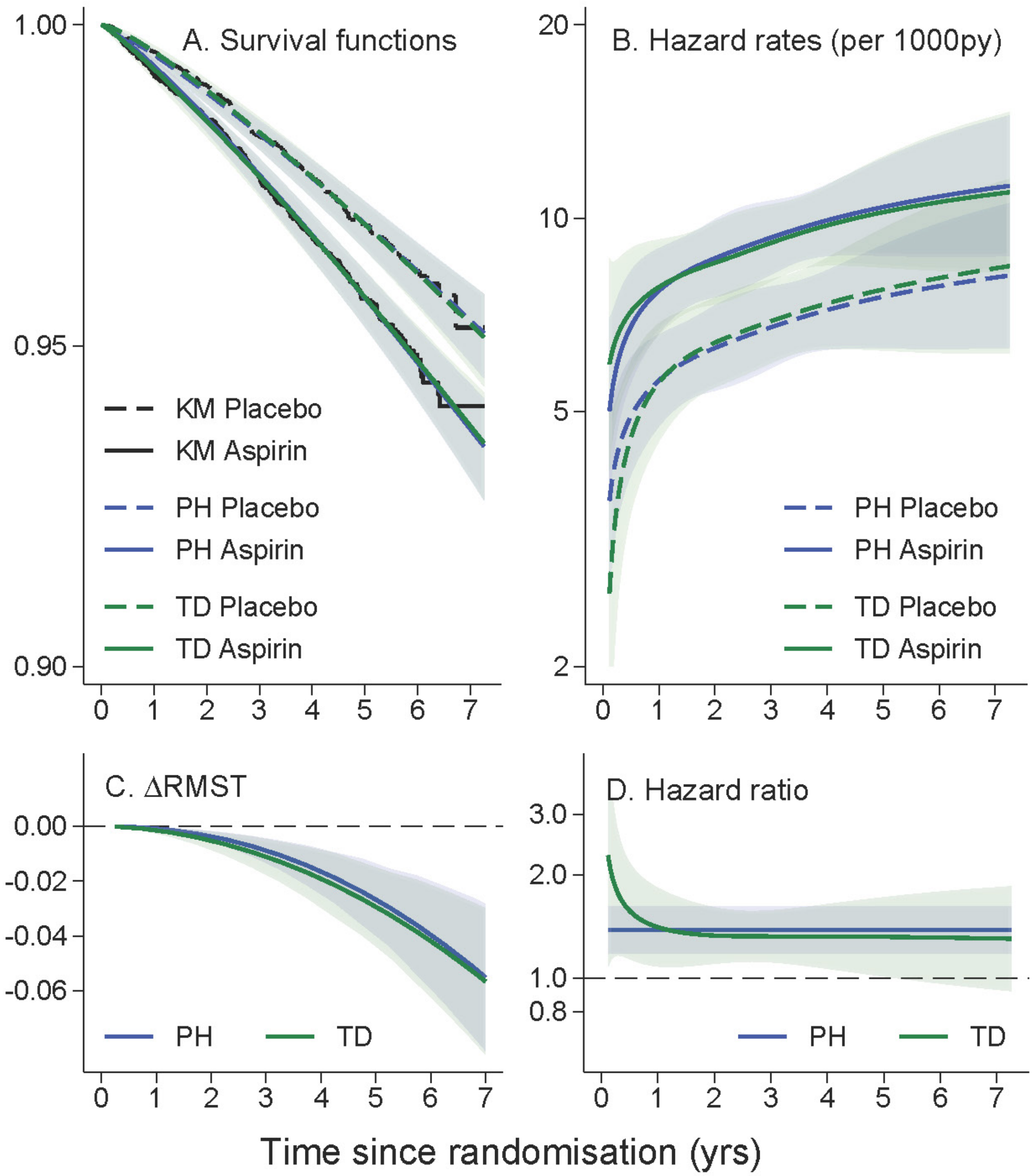

Supplementary Figure S2: Survival curves (panel A) and hazard rates (panel B) by treatment arm, and difference in RMST (ΔRMST; panel C) and HR (panel D) over time from PH and TD analysis models for the clinically significant bleeding endpoint.

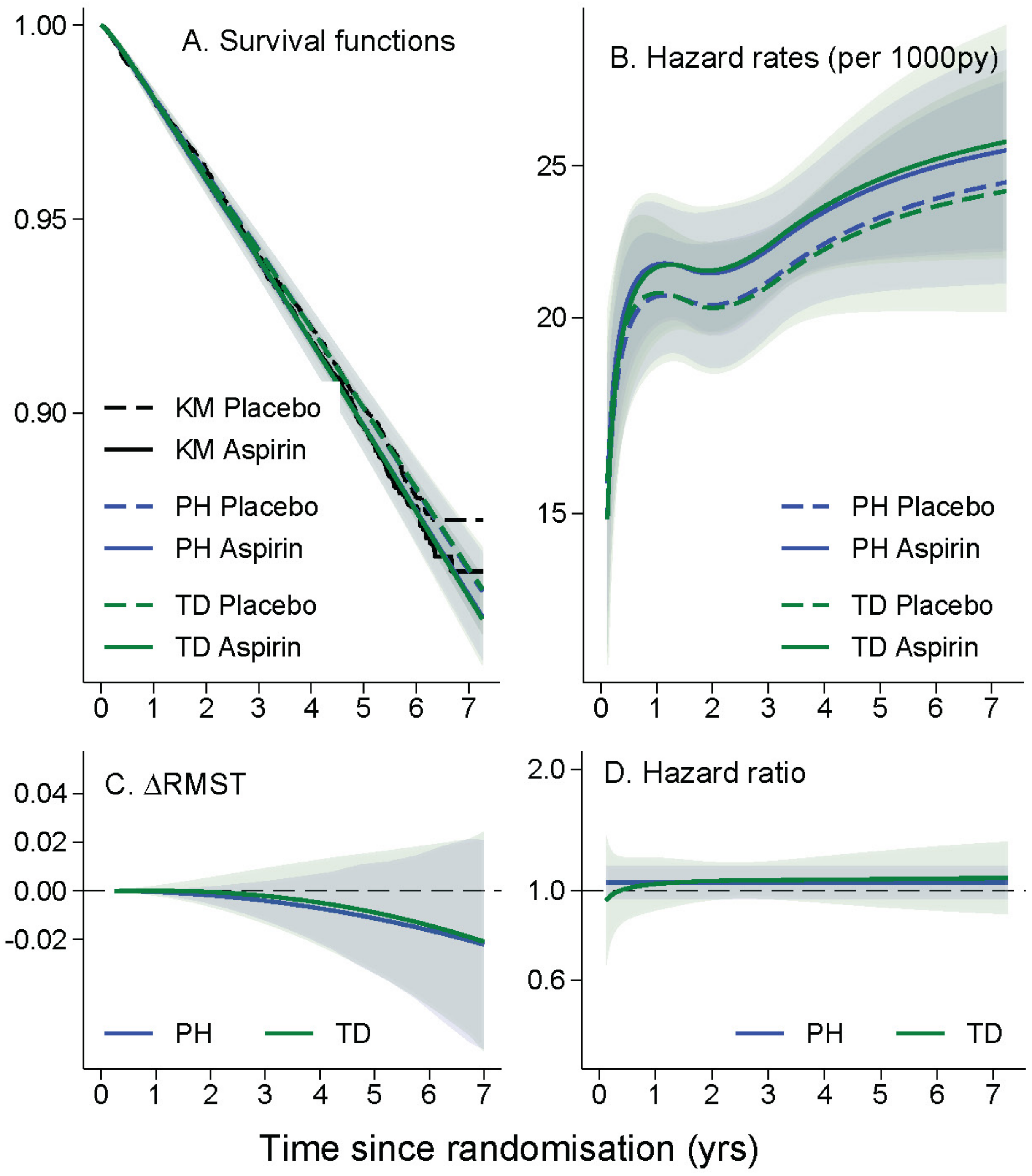

Supplementary Figure S3: Survival curves (panel A) and hazard rates (panel B) by treatment arm, and difference in RMST (ΔRMST; panel C) and HR (panel D) over time from PH and TD analysis models for the cancer incidence endpoint.

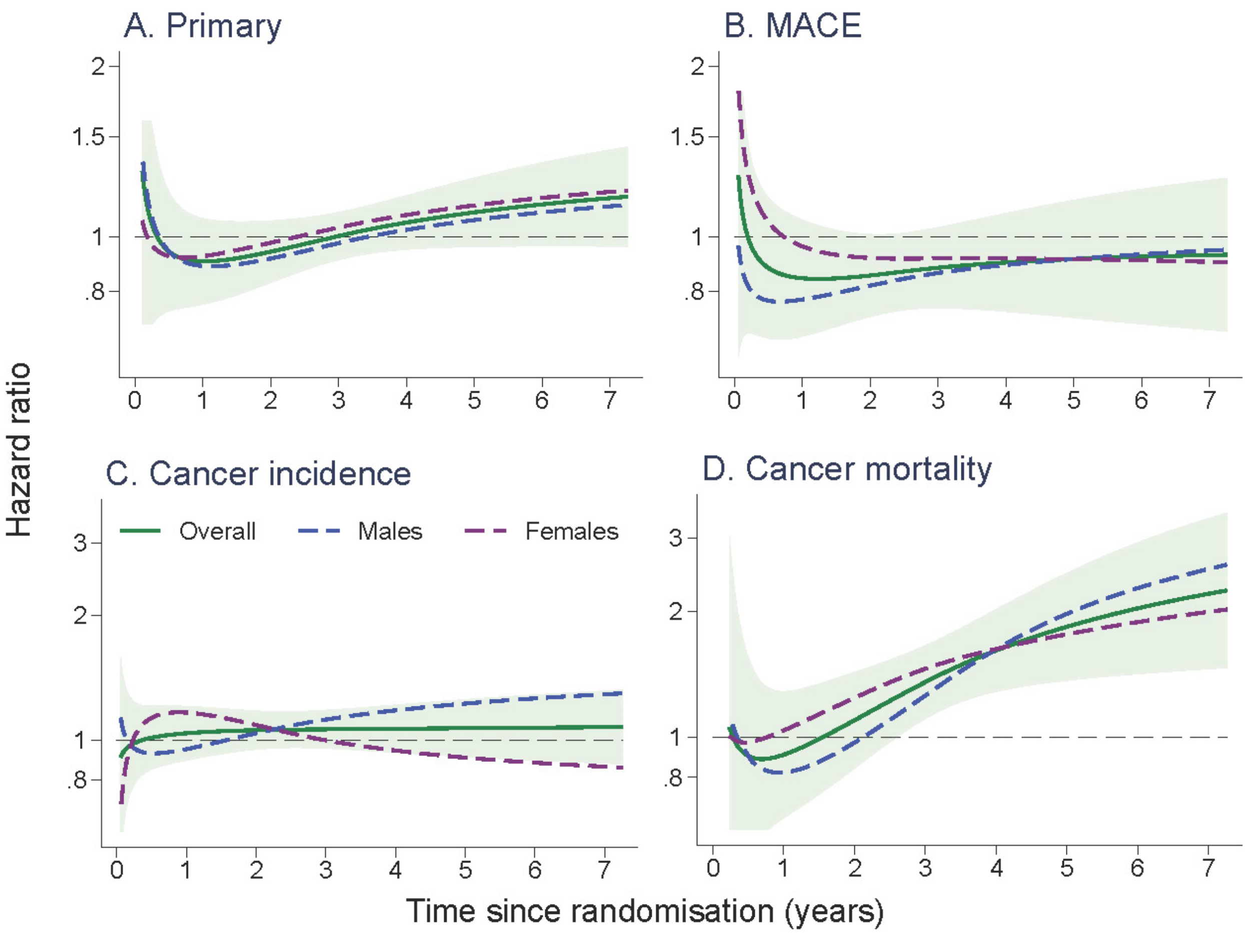

Supplementary Figure S4: Effect of binary covariate sex on the HR(t) of treatment from TD analysis models for (A) the primary, (B) MACE, (C) cancer incidence and (D) cancer mortality endpoints. The overall estimated HR(t) for treatment effect is the solid green line with the shaded green area indicating the 95% CI width. The HR(t) for treatment effect estimated from females only is indicated by a purple dashed line, and the HR(t) for treatment effect estimated from males only indicated by the blue dashed line.

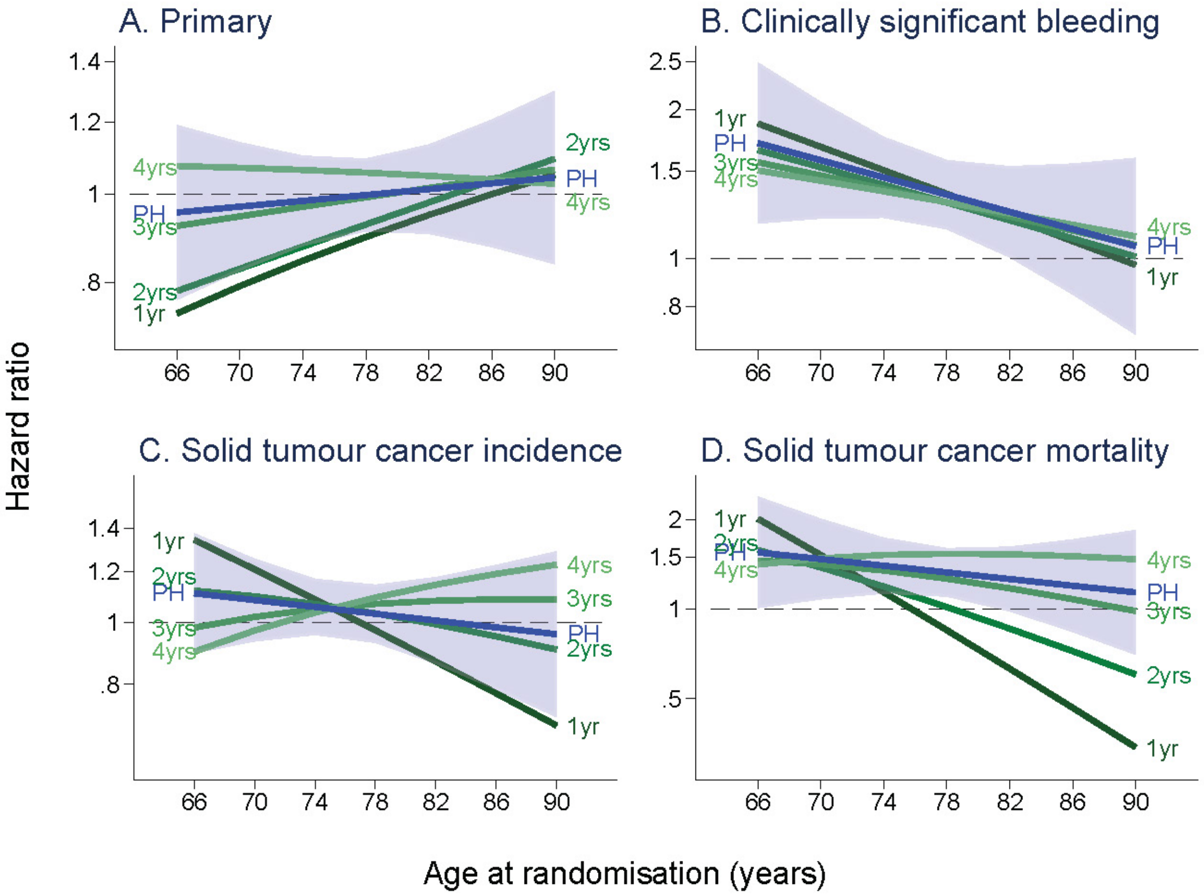

Supplementary Figure S5: Effect of covariate age at randomisation with treatment from PH and TD analysis models for (A) the primary, (B) clinically significant bleeding, (C) solid tumour cancer incidence and (D) solid tumour cancer mortality endpoints. The estimated age by treatment interaction effect from the PH model is the solid blue line. The interaction treatment effect from the TD model at yearly intervals is indicated by the green lines with color intensity decreasing over time.

Supplementary Table S1: Overall and yearly incremental treatment effect estimates for the **primary endpoint**. Estimates are from regression-based modelling approaches assuming PH (Cox, Weibull and FPM PH) or allowing for TD treatment effects (FMP TD and pseudo-observations).

	HR (95% CI)			ΔRMST (95% CI)		
	Cox PH	Weibull PH	FPM PH	FPM PH	FPM TD	pseudo-observations
Overall	1.01 (0.92, 1.11)	1.01 (0.92, 1.11)	1.01 (0.92, 1.11)	-0.006 (-0.047, 0.035)	-0.006 (-0.047, 0.035)	-0.006 (-0.047, 0.035)
0-1 years	0.87 (0.65, 1.18)	0.87 (0.65, 1.18)	0.87 (0.65, 1.18)	0.000 (-0.000, 0.001)	0.000 (-0.001, 0.001)	0.000 (-0.001, 0.001)
0-2 years	0.99 (0.83, 1.18)	0.99 (0.83, 1.18)	0.99 (0.83, 1.18)	0.000 (-0.003, 0.004)	0.001 (-0.004, 0.005)	0.001 (-0.004, 0.005)
0-3 years	0.95 (0.83, 1.08)	0.95 (0.83, 1.08)	0.95 (0.83, 1.08)	0.003 (-0.004, 0.010)	0.002 (-0.007, 0.010)	0.001 (-0.008, 0.009)
0-4 years	0.96 (0.86, 1.07)	0.96 (0.86, 1.07)	0.96 (0.86, 1.07)	0.005 (-0.007, 0.017)	0.005 (-0.009, 0.019)	0.005 (-0.010, 0.019)
0-5 years	0.99 (0.89, 1.09)	0.99 (0.89, 1.09)	0.99 (0.89, 1.09)	0.003 (-0.016, 0.022)	0.006 (-0.015, 0.027)	0.006 (-0.015, 0.027)
0-6 years	1.01 (0.92, 1.11)	1.01 (0.92, 1.11)	1.01 (0.92, 1.11)	-0.003 (-0.032, 0.027)	0.002 (-0.027, 0.032)	0.002 (-0.028, 0.032)

Supplementary Table S2: Overall and yearly incremental treatment effect estimates for the **clinically significant bleeding endpoint**. Estimates are from regression-based modelling approaches assuming PH (Cox, Weibull and FPM PH) or allowing for TD treatment effects (FMP TD and pseudo-observations).

	HR (95% CI)			ΔRMST (95% CI)		
	Cox PH	Weibull PH	FPM PH	FPM PH	FPM TD	pseudo-observations
Overall	1.38 (1.18, 1.62)	1.38 (1.18, 1.62)	1.38 (1.18, 1.62)	-0.050 (-0.075, -0.026)	-0.052 (-0.077, -0.027)	-0.053 (-0.078, -0.028)
0-1 years	1.84 (1.25, 2.70)	1.84 (1.25, 2.70)	1.84 (1.25, 2.70)	-0.002 (-0.003, -0.001)	-0.001 (-0.003, -0.000)	-0.001 (-0.003, -0.000)
0-2 years	1.56 (1.20, 2.04)	1.56 (1.20, 2.04)	1.56 (1.20, 2.04)	-0.005 (-0.008, -0.002)	-0.005 (-0.009, -0.002)	-0.006 (-0.009, -0.002)
0-3 years	1.37 (1.12, 1.68)	1.37 (1.12, 1.68)	1.37 (1.12, 1.68)	-0.008 (-0.014, -0.003)	-0.011 (-0.017, -0.004)	-0.011 (-0.017, -0.004)
0-4 years	1.41 (1.18, 1.69)	1.41 (1.18, 1.69)	1.41 (1.18, 1.69)	-0.018 (-0.027, -0.008)	-0.019 (-0.029, -0.009)	-0.019 (-0.030, -0.009)
0-5 years	1.38 (1.17, 1.63)	1.38 (1.17, 1.63)	1.38 (1.17, 1.63)	-0.027 (-0.040, -0.013)	-0.029 (-0.043, -0.015)	-0.029 (-0.044, -0.014)
0-6 years	1.38 (1.17, 1.62)	1.38 (1.17, 1.62)	1.38 (1.17, 1.62)	-0.039 (-0.059, -0.020)	-0.042 (-0.062, -0.021)	-0.042 (-0.062, -0.021)

Supplementary Table S3: Overall and yearly incremental treatment effect estimates for the **major adverse cardiovascular events (MACE)** endpoint. Estimates are from regression-based modelling approaches assuming PH (Cox, Weibull and FPM PH) or allowing for TD treatment effects (FMP TD and pseudo-observations).

	HR (95% CI)			ΔRMST (95% CI)		
	Cox PH	Weibull PH	FPM PH	FPM PH	FPM TD	pseudo-observations
Overall	0.89 (0.77, 1.03)	0.89 (0.77, 1.03)	0.89 (0.77, 1.03)	0.021 (-0.005, 0.048)	0.021 (-0.005, 0.048)	0.020 (-0.008, 0.047)
0-1 years	1.07 (0.76, 1.53)	1.07 (0.76, 1.53)	1.07 (0.76, 1.53)	-0.000 (-0.001, 0.001)	-0.001 (-0.002, 0.001)	-0.000 (-0.002, 0.001)
0-2 years	0.86 (0.68, 1.09)	0.86 (0.68, 1.09)	0.86 (0.68, 1.09)	0.002 (-0.001, 0.005)	0.000 (-0.004, 0.004)	0.000 (-0.004, 0.004)
0-3 years	0.85 (0.70, 1.03)	0.85 (0.70, 1.03)	0.85 (0.70, 1.03)	0.005 (-0.001, 0.012)	0.003 (-0.004, 0.010)	0.004 (-0.003, 0.011)
0-4 years	0.87 (0.73, 1.03)	0.87 (0.73, 1.03)	0.87 (0.73, 1.03)	0.008 (-0.002, 0.018)	0.007 (-0.004, 0.018)	0.008 (-0.003, 0.019)
0-5 years	0.88 (0.75, 1.03)	0.88 (0.75, 1.03)	0.88 (0.75, 1.03)	0.012 (-0.002, 0.027)	0.011 (-0.004, 0.027)	0.012 (-0.004, 0.028)
0-6 years	0.88 (0.76, 1.03)	0.88 (0.76, 1.03)	0.88 (0.76, 1.03)	0.017 (-0.003, 0.037)	0.016 (-0.005, 0.037)	0.017 (-0.004, 0.038)

Supplementary Table S4: Overall and yearly incremental treatment effect estimates for the **cancer incidence endpoint**. Estimates are from regression-based modelling approaches assuming PH (Cox, Weibull and FPM PH) or allowing for TD treatment effects (FMP TD and pseudo-observations).

	HR (95% CI)			ΔRMST (95% CI)		
	Cox PH	Weibull PH	FPM PH	FPM PH	FPM TD	pseudo-observations
Overall	1.05 (0.95, 1.15)	1.05 (0.95, 1.15)	1.05 (0.95, 1.15)	-0.020 (-0.061, 0.021)	-0.018 (-0.061, 0.021)	-0.019 (-0.061, 0.021)
0-1 years	0.99 (0.80, 1.22)	0.99 (0.80, 1.22)	0.99 (0.80, 1.22)	0.000 (-0.002, 0.002)	0.000 (-0.002, 0.002)	0.000 (-0.002, 0.002)
0-2 years	1.06 (0.91, 1.22)	1.06 (0.91, 1.22)	1.06 (0.91, 1.22)	-0.002 (-0.007, 0.003)	-0.001 (-0.007, 0.005)	-0.001 (-0.007, 0.005)
0-3 years	1.03 (0.91, 1.15)	1.03 (0.91, 1.15)	1.03 (0.91, 1.15)	-0.002 (-0.012, 0.008)	-0.002 (-0.013, 0.009)	-0.002 (-0.014, 0.009)
0-4 years	1.04 (0.94, 1.14)	1.04 (0.94, 1.14)	1.04 (0.94, 1.14)	-0.006 (-0.022, 0.010)	-0.005 (-0.022, 0.013)	-0.005 (-0.023, 0.010)
0-5 years	1.04 (0.95, 1.15)	1.04 (0.95, 1.15)	1.04 (0.95, 1.15)	-0.010 (-0.033, 0.013)	-0.009 (-0.034, 0.017)	-0.008 (-0.034, 0.017)
0-6 years	1.04 (0.94, 1.14)	1.04 (0.94, 1.14)	1.04 (0.94, 1.14)	-0.013 (-0.045, 0.019)	-0.012 (-0.045, 0.021)	-0.014 (-0.048, 0.019)

Supplementary Table S5: Overall and yearly incremental treatment effect estimates for the **cancer mortality endpoint**. Estimates are from regression-based modelling approaches assuming PH (Cox, Weibull and FPM PH) or allowing for TD treatment effects (FPM TD and pseudo-observations).

	HR (95% CI)			ΔRMST (95% CI)		
	Cox PH	Weibull PH	FPM PH	FPM PH	FPM TD	pseudo-observations
Overall	1.36 (1.13, 1.63)	1.36 (1.13, 1.63)	1.36 (1.13, 1.63)	-0.032 (-0.052, 0.013)	-0.029 (-0.048, 0.010)	-0.029 (-0.049, 0.010)
0-1 years	0.90 (0.47, 1.73)	0.90 (0.47, 1.73)	0.90 (0.47, 1.73)	0.000 (-0.001, 0.001)	0.000 (-0.001, 0.001)	0.000 (-0.001, 0.001)
0-2 years	1.04 (0.72, 1.50)	1.04 (0.72, 1.50)	1.04 (0.72, 1.50)	-0.000 (-0.002, 0.002)	0.000 (-0.002, 0.002)	0.000 (-0.002, 0.002)
0-3 years	1.06 (0.82, 1.38)	1.06 (0.82, 1.38)	1.06 (0.82, 1.38)	-0.001 (-0.004, 0.003)	-0.000 (-0.004, 0.004)	-0.000 (-0.005, 0.004)
0-4 years	1.20 (0.96, 1.50)	1.20 (0.96, 1.50)	1.20 (0.96, 1.50)	-0.005 (-0.011, 0.001)	-0.002 (-0.009, 0.005)	-0.002 (-0.009, 0.005)
0-5 years	1.27 (1.04, 1.55)	1.27 (1.04, 1.55)	1.27 (1.04, 1.55)	-0.012 (-0.022, -0.002)	-0.007 (-0.018, 0.003)	-0.007 (-0.018, 0.003)
0-6 years	1.36 (1.13, 1.64)	1.36 (1.13, 1.64)	1.36 (1.13, 1.64)	-0.024 (-0.038, -0.009)	-0.017 (-0.038, -0.003)	-0.018 (-0.033, -0.003)
